# Supplementary material for: A fault-intrusion-tolerant system and deadline-aware algorithm for scheduling scientific workflow in the cloud
Source: PeerJ Comput Sci. 2021 Nov 2;7:e747. doi: 10.7717/peerj-cs.747 (PMC8576554; doi:10.7717/peerj-cs.747)
Supplement: Supplemental Information 6 [file peerj-cs-07-747-s006.zip › Myproject1.1/docs/org/cloudbus/cloudsim/lists/VmList.html]

VmList


JavaScript is disabled on your browser.


- Overview
- Package
- Class
- Use
- Tree
- Deprecated
- Index
- Help

- Prev Class
- Next Class

- Frames
- No Frames

- All Classes

- Summary:
- Nested |
- Field |
- Constr |
- Method

- Detail:
- Field |
- Constr |
- Method


org.cloudbus.cloudsim.lists

## Class VmList

- java.lang.Object
- - org.cloudbus.cloudsim.lists.VmList

- Direct Known Subclasses:
  :   PowerVmList

  ---

    

  ```
  public class VmList
  extends java.lang.Object
  ```

  VmList is a collection of operations on lists of VMs.

  Since:
  :   CloudSim Toolkit 2.0

- - ### Constructor Summary

    Constructors

    Constructor and Description

    `VmList()`
  - ### Method Summary

    Methods

    | Modifier and Type | Method and Description |
    | --- | --- |
    | `static <T extends Vm>  T` | `getById(java.util.List<T> vmList, int id)` Return a reference to a Vm object from its ID. |
    | `static <T extends Vm>  T` | `getByIdAndUserId(java.util.List<T> vmList, int id, int userId)` Return a reference to a Vm object from its ID and user ID. |

    - ### Methods inherited from class java.lang.Object

      `clone, equals, finalize, getClass, hashCode, notify, notifyAll, toString, wait, wait, wait`

- - ### Constructor Detail


    - #### VmList

      ```
      public VmList()
      ```
  - ### Method Detail


    - #### getById

      ```
      public static <T extends Vm> T getById(java.util.List<T> vmList,
                             int id)
      ```

      Return a reference to a Vm object from its ID.

      Parameters:
      :   `id` - ID of required VM
      :   `vmList` - the vm list

      Returns:
      :   Vm with the given ID, $null if not found


    - #### getByIdAndUserId

      ```
      public static <T extends Vm> T getByIdAndUserId(java.util.List<T> vmList,
                                      int id,
                                      int userId)
      ```

      Return a reference to a Vm object from its ID and user ID.

      Parameters:
      :   `id` - ID of required VM
      :   `userId` - the user ID
      :   `vmList` - the vm list

      Returns:
      :   Vm with the given ID, $null if not found


- Overview
- Package
- Class
- Use
- Tree
- Deprecated
- Index
- Help

- Prev Class
- Next Class

- Frames
- No Frames

- All Classes

- Summary:
- Nested |
- Field |
- Constr |
- Method

- Detail:
- Field |
- Constr |
- Method
